# Comparison of sensory and instrumental methods for the analysis of texture of cooked individually quick frozen and fresh‐frozen catfish fillets

**DOI:** 10.1002/fsn3.737

**Published:** 2018-07-27

**Authors:** John M. Bland, Karen L. Bett‐Garber, Carissa H. Li, Suzanne S. Brashear, Jeanne M. Lea, Peter J. Bechtel

**Affiliations:** ^1^ USDA, ARS Southern Regional Research Center New Orleans Louisiana

**Keywords:** catfish, fillet, instrumental, sensory, texture

## Abstract

Catfish fillet texture is important to consumers, especially if the texture is not what the consumer expects. Therefore, it is important to be able to assure that texture quality is consistent. Texture is a humanly perceived sensory trait and can be costly to processors when texture quality is substandard. Instrumental methods of monitoring texture are much less costly over time than maintaining a sensory quality panel. The purpose of this research was to develop methods for monitoring texture quality using reliable instrumental methods. A descriptive sensory texture panel evaluated fresh‐frozen and individually quick frozen (IQF) catfish fillets and was compared to the instrumental analysis of the same cooked fish, using texture profile analysis (TPA). The TPA evaluation was more successful for identifying differences between IQF and fresh‐frozen catfish, with the most significance (*p *<* *0.02) seen for the attributes springiness, resilience, chewiness‐1, hardness‐1, and residual parameters of springiness, chewiness‐1, chewiness‐1b, and hardness‐1b. For sensory evaluation, only moisture release and moisture retention were this significant. Overall, IQF fillets were more moist and cohesive, with fresh‐frozen fillets greater in all other parameters. Predictive equations were developed for sensory texture attributes from various TPA attributes calculated from the compression–force curves generated from two compressions of a ball probe. In the fresh‐frozen catfish, sensory attributes firmness, flaky, moisture retention, and residual cohesiveness of mass had correlation coefficients (*R*) of 0.50 or greater. For the IQF catfish, all sensory attributes had an *R* of less than 0.4. The firmness sensory attribute had TPA predictor variables in both fresh‐frozen and IQF that consisted mainly of hardness, chewiness, or thickness‐related attributes. Based on results, instrumental texture of catfish should be measured before further processing, such as IQF.

## 1 INTRODUCTION

Since the peak of the U.S. catfish industry in 2003, the amount of catfish sold to processors has decreased by more than half, from 662 million pounds to 301 million pounds in 2014 (Hanson & Sites, 2015), while offshore imports of tilapia and *Pangasius* have increased dramatically. By 2011, U.S. per capita consumption of *Pangasius* alone was greater than that for U.S. grown catfish. There are a number of reasons for the decline of the U.S. catfish industry including price and availability. This has increased the importance of producing a product with consistently high quality in an efficient manner. One of the major quality attributes of fish products is the texture of the cooked products.

Texture is considered to be one of the most important quality attributes of fish and meat. It contributes to consumer acceptance and therefore marketability of the final product (Cheret, Delbarre‐Ladrat, Lamballerie‐Anton, & Verrez‐Bagnis, 2007). Fish texture is mainly dependent upon its fat and collagen content; however, muscle softening can occur as a result of myofibrillar protein degradation due to microbiological and autolysis processes initiated at fish death (Li et al., 2012). The general area of fish texture has been reviewed by several reports (Cheng, Sun, Han, & Zeng, 2014; Coppes, Pavlisko, & De Vecchi, 2002; Hyldig & Nielsen, 2001; Sigurgisladottir, Tornissen, Lie, Thomassen, & Hfsteinsson, 1997).

Fish muscle postmortem tenderization has been characterized as an unfavorable change in quality, resulting from acid lysosomal cathepsins and cytosolic neutral calcium‐activated calpains (Cheret et al., 2007). Fish texture factors include fat and collagen constituents that can be degraded by microbiological and autolysis processes caused by fish death. These processes are responsible for myofibrillar protein deterioration, leading to softening of the fish muscle (Li, Li, Hu, & Li, 2013; Li et al., 2012), and are thought to be of importance in the decrease in cutting force seen in fish fillets during refrigerated storage.

One of the problems in measuring texture is the requirement for a specific sample size to be cut from the meat to standardize the effects on texture. In poultry meat tenderness studies, a number of sample dimensions have been described (DeMan & Kamel, 1981; Lyon & Lyon, 1990, 1993, 1997; Sams, 1990). For cooked cod fillets, Segars and Johnson (1986) resorted to using a single flake to measure texture. For catfish fillets, there is the change in fillet thickness from the tail to head region and from dorsal to ventral region. Correlation between thickness or position on the fillet and instrumental texture for cooked fresh‐frozen and cooked individually quick frozen (IQF) fillets has been described, where decreasing hardness was observed from front to middle to end pieces corresponding to their decreasing fillet thickness (Li, Bland, & Bechtel, 2017). Veland and Torrissen (1999) demonstrated a variable correlation between many textural parameters and fillet thickness, dependent on the compression distance. Texture of par fried fish has been examined by Moradi, Bakar, Syed Muhamad, and Che Man (2009), who reported that the texture (adhesiveness, springiness, and cohesiveness) values of par fried black pomfret increased slightly when par fried samples were baked. Ojagh, Shabanpour, and Jamshidi (2013) evaluated the sensory properties of par fried fish products including evaluation of silver carp fish nuggets, par fried at different temperatures. Vacuum tumbling has been shown to increase tenderization of catfish fillet (Kin et al., 2011), changing the texture from that of fresh fish.

Sensory and instrumental relationships have been the subject of several publications (Drake & Gerard, 1999; Lyon, Champagne, Vinyard, & Windham, 2000; Meullenet, Lyon, Carpenter, & Lyon, 1998; Pascua, Koc, & Foegeding, 2013). Relationships between texture profile analysis (TPA) and sensory evaluation by a trained panel for cooked sweet potatoes have been described (Truong, Daubert, Drake, & Baxter, 2002). The correlation of instrumental/physical methods with sensory analysis of frozen fish was reviewed by Barroso, Careche, and Borderias (1998).

Common methods for measuring fish texture include sensory analysis, Warner Bratzler shear testing and TPA. Instrumental methods including TPA and a Warner‐Bratzler shear test were used to evaluate texture of uncooked Atlantic salmon stored on ice for up to 24 days (Veland & Torrissen, 1999). Li et al. (2017) reported the use of a 12.7‐mm (1/2 in) ball probe to determine that cooked catfish fillets were harder for fresh‐frozen than for IQF fillets. Sigurgisladottir et al. (1999) used 25‐mm cylinder, 25.4‐mm spherical probes for puncture tests, and a 3‐mm blade for shear force measurements, to evaluate the texture of raw salmon fillets. Other methods have been reported to analyze the texture of fish fillets (Ashton, Michie, & Johnston, 2010; Cheng & Opara, 2013; Jiang, Wang, van Santen, & Chappel, 2008; and Nakayama, Hatae, Kasai, & Ooi, 2017). Other interesting texture studies conducted on other species include catfish (Hallier, Chevallier, Serot, & Prost, 2007; Lingqiao, Chenglong, Jingjing, & Dapeng, 2014), giant grenadier (Crapo, Himelbloom, Pfutzenreuter, & Lee, 1999), and carp (Vacha et al., 2013).

Most commercially processed aquaculture produced catfish fillets are treated with phosphate and frozen prior to distribution. The most common freezing process is individually quick frozen fillets (IQF). Most commercially available polyphosphates are blends of sodium tripolyphosphate and hexametaphosphate, which are widely used in the meat and seafood industry to preserve food quality. Phosphates have been used to retain muscle juiciness and reduce drip loss and cooking loss during cooking and freezing. During frozen storage, myofibrillar proteins readily denature and lose much of their water binding capacity (Sheard, Nute, Richardson, Perry, & Taylor, 1999; Woyewoda & Bligh, 1986). When properly used, polyphosphate treatment has little flavor and retards oxidative deterioration of muscle by chelating heavy metal ions (Lampila, 1993). Besides retaining natural muscle moisture and preventing lipid oxidation, the ability of increasing thermal stability of proteins and imparting cryoprotection is also beneficial to preserving quality of food (Etemadian, Shabanpour, Sadeghi Mahoonak, Shabani, & Alami, 2011). The type of phosphate used for injection into catfish fillets was evaluated by Kin et al. (2010). Polyphosphate treatment increases juiciness and tenderness of meat and seafood products due to the weakened muscle structure (Griffiths & Wilkinson, 1978; Klose, Campbell, & Hanson, 1963; Sheard et al., 1999). Cooked IQF catfish fillets were not as hard as fresh‐frozen fillets (Li et al., 2017).

Currently, the measurement of catfish texture using instrumental means has not been related to descriptive sensory texture evaluation. The research objectives in this study were to contrast the texture characteristics of cooked catfish fillets using both TPA and a trained sensory panel. An additional objective was to compare the textural characteristics of fresh‐frozen catfish fillets with commercially available IQF catfish fillets that were treated with phosphate prior to freezing.

## 2 MATERIALS AND METHODS

### 2.1 Fish samples

The fresh‐frozen sample consisted of 34 catfish obtained from an experimental pond in Stoneville, MS and the IQF sample was 60 catfish obtained from a commercial processing plant in Alabama. Both types were a mixture of *Ictalurus punctatus* and *I. punctatus* × *I. furcatus* hybrids.

The fresh‐frozen fish were from a single pond but included a variety of families resulting from multiple spawns. They were reared as fry in separate family tanks for about 10 months and fed a fingerling diet (35% protein, Fishbelt Feed) to satiation once daily, they were then tagged with individually coded pit tags on the left fillet, and stocked communally in an earthen pond. They were fed a commercial foodfish diet (32% Delta Western) daily from April through October of 2015. They were then fed once a week until processing on 14 January 2016. With an average age of 592 ± 9 days and average weight of 771 ± 129 g, the fish were seined from the pond and held in a cement Raceway overnight at 11–16°C (52–61°F). Fish were electrically stunned by a 40 V electric pulse (Sylvesters, Inc., Louisville, MS, USA), deheaded (Baader 166; Baader North America, Indianola, MS, USA), gutted by hand, filleted (Baader 184), and trimmed by hand. Both fillets from each fish were weighed and stored individually in a ziplock storage bag. All fillets were quickly placed in a −20°C freezer overnight, before being transported on ice to the research facility and stored at −20°C.

Individually quick frozen individually quick frozen fish were transported to a commercial processing plant by truck (<15 miles) from multiple ponds (within 4 miles apart). They had been fed a commercial diet (32% AL Catfish Feed mill). After netting the previous night, they were socked, loaded, and shipped within a 2‐hr span on the morning of processing. Fish were weighed, and those from 600 to 900 g were used for the study. Fillets were processed, including phosphate treatment (dip), and IQF in a mechanical blast freezer. Both fillets from each catfish were collected and stored individually in a ziplock storage bag, transported on ice to the research facility, and stored at −20°C.

For all fish, the right fillet was used for sensory evaluation and the left fillet used for instrumental texture profile analysis. Sensory and instrumental analysis was performed within 6 months.

### 2.2 Preparing fish samples

The fish samples were thawed in the refrigerator overnight. On the day of the panel session, the fish fillets were wrapped in foil that was perforated to allow steam to escape. They were baked in convection ovens (Wisco Industries, Inc.) at 149°C (300°F) to an internal temperature of 74°C (165°F).

### 2.3 Sensory evaluation

Eleven panelists trained in descriptive analysis methods (Meilgaard, Civille, & Carr, 2007) evaluated seven texture attributes: flaky, springiness, firmness, moisture release, fibrous, moisture retention, and cohesiveness of mass (Table 1) using previously established texture intensity scales (Chambers & Robel, 1993). There were 16 sessions to present and practice the individual texture scales. The eleven panelists were broken up into four groups so that each group would receive portions from one fish fillet that was taken from the thickest part of the fillet. This gave each panelist a big enough piece of fish to adequately evaluate the seven texture attributes. Samples were served 20 min apart to allow panelists to evaluate the sample and cleanse their mouth with reverse osmosis filtered water and unsalted crackers. Fish samples were placed in warmed, glass, custard bowls that were placed in foam bowls to keep the sample warm during evaluation.

**Table 1:** Sensory texture attributes with definitions and intensity references

Attribute	Definition	References
Cohesiveness of mass	The degree to which chewed sample (at 10 to 15 chews) holds together in a mass (forms a ball)	3.0 = Raw, button mushroom, 1/2″ cube
		5.0 = Hebrew National all beef hot dog, boiled 4 min, 1/2″cube
		8.0 = Chicken Breast, microwaved, 1/2″ cube
Fibrous	The perception of filaments or strands of muscle tissue during mastication	2.0 = Ball Park All Beef hot dog, boiled 4 min, 1/2″ cube
		3.0 = Mariani Dried Mango, 1/2″ cube
		5.0 = Boar's Head Deli Turkey, 1/2″ cube
		7.0 = Starkist Solid Albacore canned tuna (water packed), 1/2″ cube
		8.0 = Dole Pineapple chunk (in 100% pineapple juice), 1/2″piece
		10.0 = Chicken Breast, microwaved, 1/2″ cube
Firmness	Amount of force required to bite through the flesh when the sample is placed between molar teeth	1.0 = Ball Park All Beef hot dog, boiled 4 min, 1/2″ cube
		2.0 = Hard‐boiled egg white, 1/2″ cube
		4.0 = Land o Lakes yellow American pasteurized cheese, 1/2″ cube
		5.0 = Hebrew National all beef hot dog, boiled 4 min, 1/2″cube
		7.0 = Boar's Head Deli Turkey, 1/2″ cube
		10.0 = Chicken Breast, microwaved, 1/2″ cube
Flaky (visual)	The ease of breaking the fish into small pieces with a fork	2.0 = Boar's Head Deli Turkey, 1/2″ cube
		5.0 = Starkist Solid Albacore canned tuna (water packed), 1/2″ cube
		7.0 = Bumble Bee Fancy Lump crab meat, 1/2″ cube
Moisture release (juicy initial)	Bite with molars then evaluate the amount of liquid released when the sample is placed on tongue and pressed to the roof of the mouth	2.0 = Oscar Meyer All Beef hot dog, boiled 4 min, 1/2″ cube
		5.0 = Hebrew National all beef hot dog, boiled 4 min, 1/2″cube
		6.0 = Boar's Head Deli Turkey, 1/2″ cube
		11.0 = Sliced orange, 1/2″ cube
Moisture retention (juicy mid point)	Amount of liquid observed in the mass after 5 chews with the molar teeth	4.0 = Boar's Head Deli Turkey, 1/2″ cube
		5.0 = Hebrew National all beef hot dog, boiled 4 min, 1/2″cube
		7.0 = Ball Park All Beef hot dog, boiled 4 min, 1/2″ cube
Springiness	The degree to which sample returns to original shape or the rate with which sample returns to original shape	2.0 = General Mills fruit chew, 3 pieces
		3.0 = Land o Lakes yellow American pasteurized cheese, 1/2″ cube
		5.0 = Hebrew National all beef hot dog, boiled 10 min, 1/2″cube
		9.5 = Kraft Miniature Marshmallow, 3 pieces

### 2.4 Texture profile analysis

**Figure 1:**
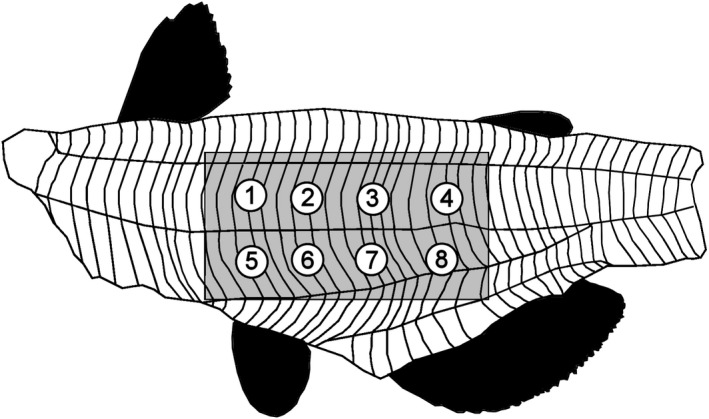
Depiction of eight positions on the fillet used for instrumental texture profile analysis. The rectangle area was cut from the fillet for cooking before texture analysis

**Figure 2:**
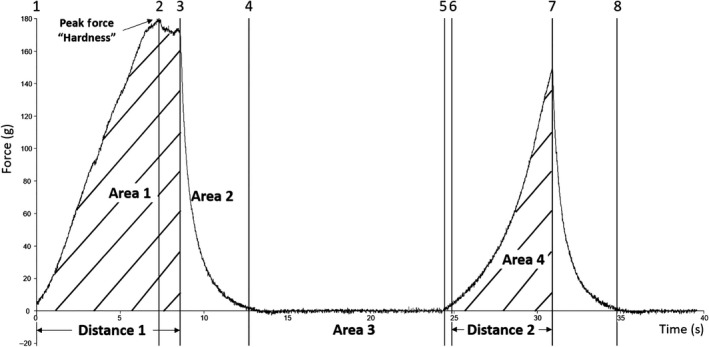
TPA force‐time graph showing anchor points used to measure attributes. This was a nonrepresentative sample that showed a separation between anchors 2 and 3. TPA, texture profile analysis

Fillets were thawed overnight in a refrigerator, weighed, and a middle rectangle, of dimensions 8.3 cm × 6.2 cm (head to tail × dorsal to ventral), was cut from the fillet. The fillet section was weighed, and a temperature probe (1/16″ diameter, Pro‐Series Needle Probe, cat # TX‐1002X‐NP), connected to a DOT (ThermoWorks, American Fork, UT, USA), was inserted into the center of the fillet. The fillet was wrapped in aluminum foil that was perforated to allow steam to escape, placed on a cooking pan, and baked in a professional convection oven at 149°C (300°F) to an internal temperature of 74°C (165°F) (approx. 10 min). The fillet was cooled to a surface temperature of approx. 30°C (86°F) (approx. 12 min) and placed on the heavy duty platform of the texture analyzer (TA.XT plus; Texture Technologies, Hamilton, MA). Parameters for texture analysis were as follows: texture profile analysis sequence of two compressions, 30 kg load cell, 1/2″ diameter ball probe (TA‐18), trigger force = 5 g, strain = 50%, pre‐test speed = 3 mm/s, test speed = 1 mm/s, post‐test speed = 1 mm/s, pause time between cycles = 5 s. The ball probe was selected instead of the TPA‐recommended flat probe or compression plates, so multiple points on the fillet could be measured without the need for cutting a perfectly sized sample. Eight points (four on the dorsal side and four on the ventral side of the mid‐ridge, 1.8 cm apart) on each fillet were tested (Figure 1). Force‐time graphs (Figure 2) for each test point were analyzed by an Exponent 32 software macro that determined the thickness of the fillet before and after compression, the maximum force of both compressions, the compression upstroke and downstroke energy, or work, as measured by area. Six texture attributes and fillet thickness were calculated by the formulae given in Table 2.

**Table 2:** Texture profile analysis attributes, with formula and description

Attribute	Formula^a^	Description
Adhesiveness	Area 3	Negative work at end of decompression
Chewiness‐1	Hardness‐1 * Cohesiveness * Springiness	Work required to chew sample to a state ready for swallowing
Cohesiveness	Area 4/Area 1	2nd compression work relative to 1st compression work
Hardness‐1	Force at anchor 2	Maximum force of a 50% compression
Resilience	Area 2/Area 1	Decompression work relative to compression work
Springiness	Distance 2/Distance 1	Relative recovery from 1st compression
Thickness‐1	2 * Distance 1	Fillet thickness – twice the 50% compression distance
Chewiness‐1b	Hardness‐1b * Cohesiveness * Springiness	Calculated with Hardness‐1b
Chewiness‐2	Hardness‐2 * Cohesiveness * Springiness	Calculated with Hardness‐2
Hardness‐1b	Area 1	Compression work
Hardness‐2	Maximum force of 2nd compression (at anchor 7)	Resistive force of 2nd compression
Thickness‐2	2 * Distance 2	Fillet thickness after first compression

*Notes*. Gray rows signify extended TPA attributes.

a See Figure 2 for formula descriptors.

In addition to the standard TPA attributes described above, a number of modified attributes were also calculated to determine whether they might have an enhanced correlation with sensory attributes. It has been noted that in many instances, the metric of peak force does not adequately replicate the energy experienced by consumers. And researchers should understand that consumers' judgments of hardness can be more nuance than a simple peak force metric and in some instances might be able to attain better correlations with the downstroke area of work (Johnson, 2014). Therefore, we have included the hardness‐1b attribute calculated in this manner. As shown in Table 2, this and several other modified attributes are shown, with their method of calculation.

Because of a variable dependency of many of the attributes on the thickness of the fillet, all attributes having a thickness correlation were modified to remove the thickness contribution (see Table 3 for equations). This is equivalent to obtaining the residual from the linear regression function between the named attribute and thickness.

**Table 3:** Modified TPA and sensory attributes, to remove contribution of thickness from original attribute

Attribute	Formula^a^
Residual chewiness‐1	Chewiness − (8.215 * Thickness‐2 − 17.386)
Residual chewiness‐1b	Chewiness‐1b − (9.807 * Thickness‐2 − 54.626)
Residual cohesiveness	Cohesiveness − (0.005 * Thickness‐2 + 0.421)
Residual hardness‐1b	Hardness‐1b − (26.845 * Thickness‐2 − 138.455)
Residual springiness	Springiness − (1.008 * Thickness‐2 + 60.275)
Residual cohesiveness of mass	Cohesiveness of mass − (0.147 * Thickness‐2 + 4.332)
Residual firmness	Firmness − (0.151 * Thickness‐2 + 2.531)
Residual flaky	Flaky − (−0.159 * Thickness‐2 + 6.546)

*Notes.* Gray rows signify sensory attributes.

a Based on the linear regression residual.

### 2.5 Statistical methods

Analysis of variance (ANOVA) to compare IQF and fresh‐frozen fish texture sensory and TPA parameters was accomplished with SAS Enterprise Guide, v. 7.1 using Proc Mixed. Means of repeated‐measures were averaged for each fish, and the individual fish means were inserted in the ANOVA. Correlation coefficients and predictor variables were generated with SAS‐JMP Pro, v. 13 using Stepwise regression. Overfitting of the model was avoided by setting the “stopping rule” value at 0.05 and using only the forward direction. Also, the adjusted R^2^ was used according to Babyak (2004). Following the main effects regression, stepwise was run again to add the squared and cross products to the equation from the main effects that qualified to enter the model during the original analysis. Root mean square errors (RMSE), coefficients of determination (*R* ^2^), and correlation coefficients (*R*) were used to indicate the significance of the model.

## 3 RESULTS AND DISCUSSION

### 3.1 Comparison of IQF processed catfish with fresh‐frozen catfish texture properties

**Figure 3:**
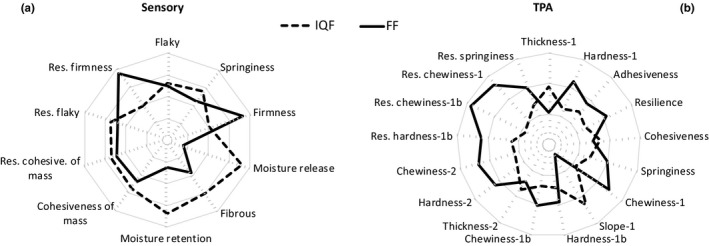
Radar graph of the relative means of the (a) sensory attributes, and (b) instrumental texture profile analysis attributes

Analysis of variance using mixed models compared the data of IQF processed catfish with fresh‐frozen catfish because the data had some missing values and were not balanced. Means of each fish were analyzed to remove the error around the repeated measures for each fish. Table 4 includes the means, standard deviations, and statistical differences between fresh‐frozen and IQF processed catfish. Figure 3a presents the sensory data on a relative scale to highlight the comparison of the difference of means between fresh‐frozen and IQF. Fresh‐frozen catfish were significantly less intense (Pr > F ≤ 0.05) in moisture release and moisture retentions. This indicates that IQF fish release more moisture upon first bite and during chewing than fresh‐frozen fish. This is most probably due to the added water and water holding ingredients such as phosphates added in the IQF process (Lampila, 1993). The fresh‐frozen catfish was markedly (Pr > F of 0.09) more firm that the IQF catfish, likewise, probably due to added water and water holding ingredients or could be due to the polyphosphate injectors during IQF processing that may tenderize the fillets. When the sensory firmness attribute was standardized for thickness (residual firmness), the fresh‐frozen catfish fillet was significantly more firm. Li et al. (2017) observed that IQF fish was less hard than fresh fish. The IQF catfish was markedly (Pr > F of 0.07) more intense in fibrous texture during chewing. This indicates that the IQF treatment causes the muscle fibers to be more pronounced during chewing than untreated frozen fish, which contradicts what Hale and Waters (1981) say about toughening of fibers due to shrinkage and drip loss, which should be less in IQF catfish. The added moisture to the IQF catfish results is fillets with more moisture on first bite and during chewing. It also, tends to be less firm.

**Table 4:** Means and standard deviations of sensory texture attributes

Attribute	IQF		Fresh‐frozen		Pr > F
	Mean	STD	Mean	STD	
Cohesiveness of mass	5.98	0.73	5.87	0.74	0.4781
Fibrous	5.33	0.77	5.03	0.75	0.0656
Firmness	4.05	0.92	4.39	0.96	0.0898
Flaky	4.82	0.92	4.79	0.80	0.8646
Moisture release	5.77 a	1.13	4.99 b	1.10	0.0016
Moisture retention	5.44 a	0.81	4.88 b	0.78	0.0016
Springiness	3.09	0.85	3.00	0.68	0.6078
Residual cohesiveness of mass^c^	0.025	0.73	−0.043	0.70	0.6616
Residual firmness^c^	−0.141 b	0.91	0.248 a	0.92	0.0497
Residual flaky^c^	0.030	0.91	−0.052	0.78	0.6635

*Notes.* IQF, individually quick frozen.

^a,b^indicates that means, within a row, are significantly different (*p *<* *0.05) and order of values.

c Attributes corrected for contribution from fillet thickness.

**Figure 4:**
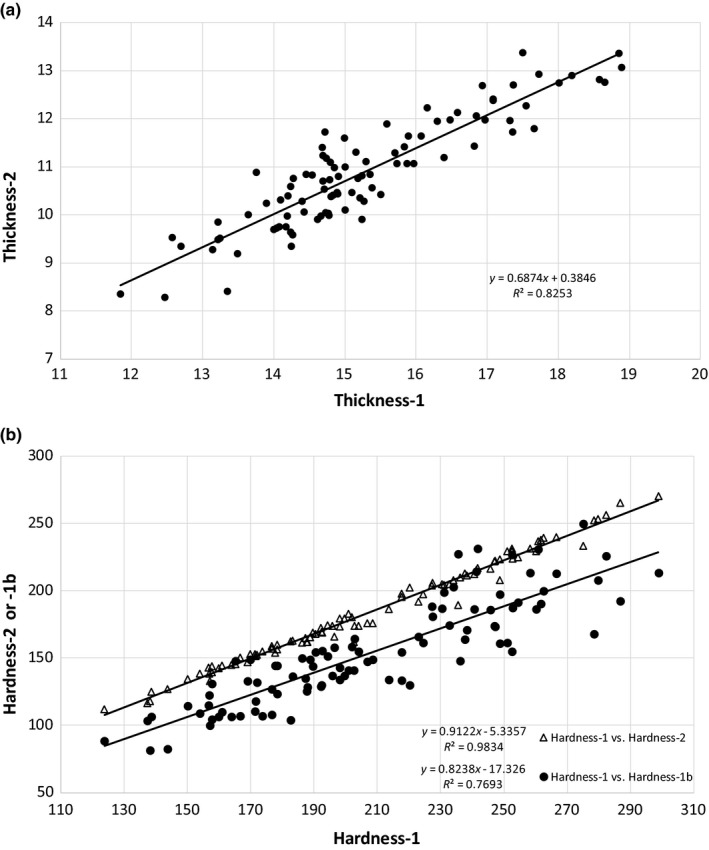
Comparison of TPA parameters, (a) thickness‐1 with thickness‐2, and (b) hardness‐1 with hardness‐2 or hardness‐1b. TPA, texture profile analysis

Comparing the TPA data of the IQF and fresh‐frozen catfish (Table 5 and Figure 3b) shows that the thickness of the IQF catfish was significantly greater than the fresh‐frozen before the first compression (thickness‐1). The thickness measurement is confounded between the IQF treatment and the source of catfish as the experimental design did not account for this. At the second compression, the thickness‐2 was not statistically different between treatments. This was likely due to the disruption of cells (Karales, 2001) after the first compression that leaves the thickness more uniform at the second compression. A comparison of thickness‐1 and thickness‐2 (Figure 4a), showed a good correlation, with an *R* ^2^ of 0.83. The first compression was found to cause an approximately 15% reduction in thickness.

**Table 5:** Means and standard deviations of texture profile analysis (TPA) data

Attribute	IQF		Fresh‐frozen		Pr > F
	Mean	STD	Mean	STD	
Adhesiveness	−1.13	0.35	−1.17	0.28	0.5530
Chewiness‐1	68.61 b	16.86	78.07 a	18.50	0.0134
Chewiness‐1b	50.857	15.70	54.350	12.66	0.2706
Chewiness‐2	61.15 b	15.27	69.18 a	17.83	0.0235
Cohesiveness	0.48 a	0.02	0.47 b	0.03	0.0270
Hardness‐1	200.34 b	42.19	220.51 a	34.81	0.0201
Hardness‐1b	151.22	42.45	158.15	29.31	0.4011
Hardness‐2	178.06 b	38.16	194.68 a	34.05	0.0379
Resilience	21.73 b	2.84	23.50 a	1.79	0.0015
Springiness	69.77 b	2.51	73.84 a	2.65	<0.0001
Thickness‐1	15.73 a	1.53	14.47 b	1.09	<0.0001
Thickness‐2	10.99	1.25	10.69	0.90	0.2164
Residual chewiness‐1^c^	−4.326 b	13.28	7.634 a	15.87	0.0002
Residual chewiness‐1b^c^	−3.713 b	12.05	6.552 a	15.79	0.0006
Residual cohesiveness^c^	0.004 a	0.02	−0.007 b	0.03	0.049
Residual hardness‐1b^c^	−5.462 b	25.34	9.639 a	12.76	0.0016
Residual springiness^c^	−1.585 b	2.01	2.798 a	2.39	<0.0001

*Notes*. IQF, individually quick frozen.

^a,b^indicates that means, within a row, are significantly different (*p *<* *0.05) and order of values.

c Attributes corrected for contribution from fillet thickness.

**Figure 5:**
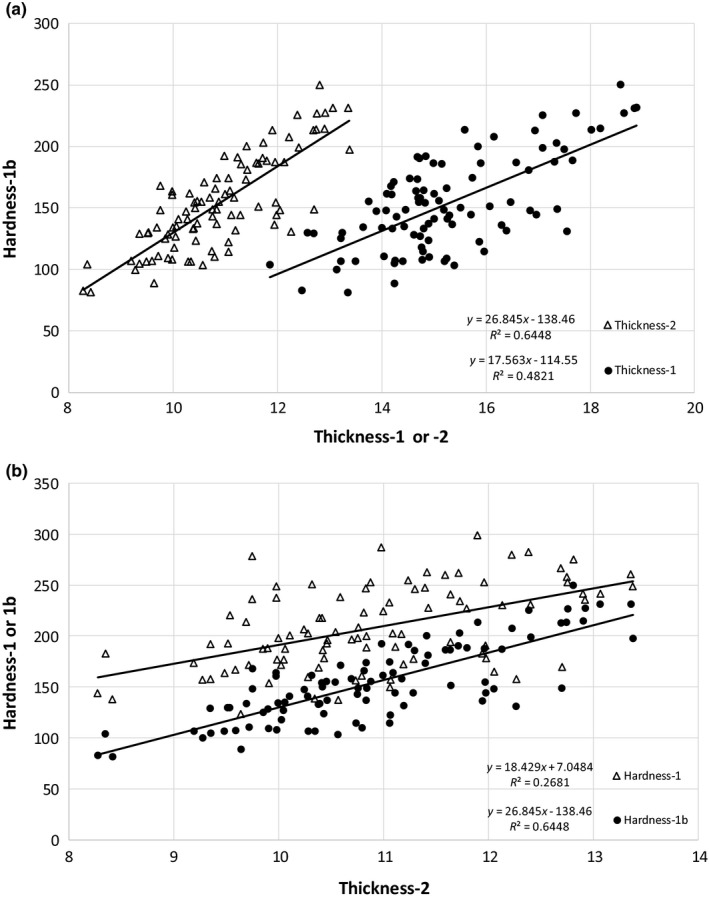
Comparison of (a) thickness and (b) hardness parameters for hardness–thickness correlation

**Figure 6:**
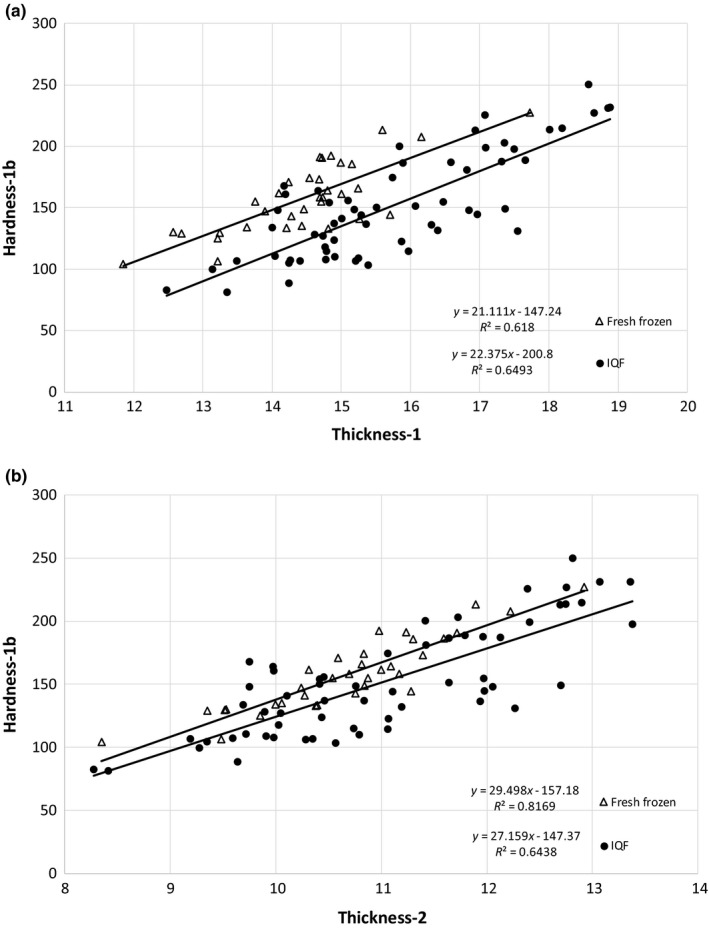
Comparison of the hardness–thickness correlation of IQF and fresh‐frozen (FF) fillets by the (a) thickness‐1 and (b) thickness‐2 parameter. IQF, individually quick frozen

Hardness‐1, which is a measure of force to compress the fish between the ball probe and plate during the first compression, was significantly greater for the fresh‐frozen catfish than the IQF catfish (Table 5). The tenderization that occurred during the injection of water solution, likely contributed to less force required for compression (Kin et al., 2011). The force during the second compression (hardness‐2) was greater in the fresh‐frozen catfish, also. Similarly, release of remaining added water could have contributed to less force required in the IQF catfish during the second compression. A comparison between the hardness‐1 parameter and either hardness‐2 or hardness‐1b is shown in Figure 4B, with both comparisons giving a similar slope. Hardness‐2, from the second compression forces, showed very good correlation, with an *R* ^2^ of 0.98. The alternative hardness parameter, hardness‐1b, derived from the area under the downstroke (upslope of the first compression curve), also had a good correlation, with an *R* ^2^ of 0.77. Figure 5A demonstrates the relationship between hardness‐1b and thickness, where increasing fillet thickness resulted in greater fillet hardness, but also shows thickness‐1 to have a lower *R* ^2^ (0.48) than thickness‐2 (0.64). Even though the two catfish processes were not significantly different for hardness‐1b (Table 5), Figure 5b shows hardness‐1b to have a greater *R* ^2^ (0.64) than hardness‐1 (0.27) in their correlation with thickness‐2. In a comparison of IQF and fresh‐frozen catfish (Figure 6), when thickness‐1 is used for the correlation (Figure 6a), both IQF and fresh‐frozen catfish have an *R* ^2^ of about 0.6. When thickness‐2 is used for the correlation (Figure 6b), fresh‐frozen catfish has a higher *R* ^2^ of 0.82 vs 0.64 for the IQF catfish. However, when using thickness‐2, the separation between the regression lines of fresh‐frozen and IQF catfish was reduced, as compared to the thickness‐1 regression lines (more pronounced at the lower thicknesses). Standardizing hardness‐1b (area measurement) to thickness (residual hardness‐1b) changed it to be significantly greater in the fresh‐frozen catfish, even more pronounced than the results of the force measurement for the first (hardness‐1) and second (hardness‐2) compressions (Table 5). Thickness of the fillet needs to be taken into consideration, as stated by Veland and Torrissen (1999).

The resilience value was also greater in the fresh‐frozen catfish. However, the cohesiveness value was slightly greater in the IQF catfish, indicating that it remains intact better than the fresh‐frozen fish during compression. The residual cohesiveness was also greater in the IQF catfish. Springiness was greater in the fresh‐frozen catfish than the IQF, indicating that fresh‐frozen could spring back upon compression better than the IQF. Residual springiness that was standardized to the fillet thickness was also greater in the fresh‐frozen catfish. Although the sensory texture attribute, springiness (Table 4), did not result in a significant difference, the TPA attribute, springiness, demonstrated fresh‐frozen catfish spring back upon compression better that the IQF catfish, indicating that the added water and added water holding compounds resulted in the flesh being less springy. The chewiness‐1 value was greater in the fresh‐frozen fish (Table 5). This indicated that fresh‐frozen fish takes longer to chew and has slower break down during chewing. When chewiness‐1 was standardized to fillet thickness (residual chewiness‐1), it was also greater in the fresh‐frozen catfish fillet. Chewiness‐1b was not significantly different between the two fish processes. But residual chewiness‐1b was greater in fresh‐frozen catfish fillet.

### 3.2 Predicting sensory texture attributes from various TPA attributes

Stepwise multiple regression was used to create models for calculating sensory texture, predicted from the TPA compression curves. Montejano, Hamann, and Lanier (1985) reported that multiple parameters from the TPA are needed to represent one sensory texture attribute. Therefore, we utilized multiple regression procedures to predict sensory quality. The IQF data and the fresh‐frozen data resulted in better correlations if the two data sets were analyzed separately. When the data sets were combined, the *R* ^2^s were all less than half those found for the separate sets (data not shown). It makes sense to calculate the predictions separately as they are two different catfish products. Table 6 lists for the fresh‐frozen catfish, the intercepts, TPA predictor variables, and the corresponding coefficients, along with the regression parameters. The *R* ^2^s ranged from 0.19 to 0.30. All RMSEs, except for moisture release, are less than 1.0. Moisture retention had the greatest *R* ^2^ at 0.30, which explained 30% of the variation in the model. The predictors included thickness‐2, residual hardness‐1b, and chewiness‐1b, in which all predictors explained 7% to 10% of the variation. The cross‐products in this equation were (thickness‐2 × residual hardness‐1b), (residual hardness‐1b × residual hardness‐1b), and (thickness‐2 × thickness‐2). Pascua et al. (2013) commented that sensory attributes such as juiciness or moisture release would be less predictable with TPA parameters, while firmness should be easier to predict, because the mechanical properties for firmness between sensory and TPA are similar. Firmness was found to have a similar *R* ^2^ (0.28) to that of moisture retention. The predictors were thickness‐1 and hardness‐1, with a cross‐product of (hardness‐1 × hardness‐1). This was similar to that reported by Morkore and Einen (2003) with smoked salmon, where hardness‐1 and hardness‐2 correlated best with sensory firmness. Flaky, with a similar *R* ^2^ of 0.27, also had hardness‐1 as its predictor, exclusively. Residual cohesiveness of mass and its precursor, cohesiveness of mass, also had similar *R* ^2^s or 0.27 and 0.24, respectively. Both have chewiness‐1b and thickness‐2 predictors, with the addition of cohesiveness added to the residual cohesiveness of mass. The other two residuals, flaky and firmness, both had a lower *R* ^2^ than its precursor nonresidual. However, residual flaky had a very similar predictor to flaky, having hardness‐2, exclusively. The low correlation of several of the equations may be due to the heterogeneousness of fish muscle and that the TPA and sensory are not measuring texture in a similar manner (Hyldig & Nielsen, 2001). In addition, when fish is cooked the collagenous myocommata dissolves allowing the myotomes to slide by each other during compression causing instrumental hardness to be less (Hyldig & Nielsen, 2001). During sensory evaluation of hardness, the panelist have the mental ability to compensate for this slippage.

**Table 6:** Fresh‐frozen fish equations that predict sensory texture from texture profile analysis (TPA) attributes

Sensory attribute	Intercept	TPA predictor variable and coefficients	RMSE	*R* ^2^	*R*
Cohesiveness of mass	1.758	−0.0073(C1b)+0.426(T2)+0.0274(C1b × T2) −0.349^b^(T2 × T2)	0.646	0.240	0.490
Fibrous	6.955	−0.725(A)‐0.118(R)	0.694	0.136	0.369
Firmness	−0.305	0.322^b^(T1) −0.001^b^(H1)+0.0003^b^(H1 × H1)	0.814	0.280	0.530
Flaky	7.533	−0.0124^a^(H1)	0.680	0.270	0.520
Moisture release	5.126	−0.0334^b^(H1r)+0.001(H1 × H1)	1.014	0.210	0.400
Moisture retention	1.301	0.354^b^(T2) −0.0247(H1br)+0.014(T2 × H1br) −0.0004(H1br × H1br) −0.0723(T2 × T2)	0.648	0.302	0.549
Springiness	3.437	−0.094^b^(Sr) −0.0309^b^(Sr × Sr)	0.615	0.190	0.430
Residual cohesiveness of mass	−10.05	12.69^b^(Co) −0.048^b^(C1b)+0.62^b^(T2) −14.73(Co × T2)+0.073^b^(C1b × T2) −0.736^a^(T2 × T2)	0.590	0.270	0.520
Residual firmness	−3.723	0.303(T1) −0.0114(C1b) +0.0018(C1b × C1b) −0.062(T1 × T1)	0.870	0.097	0.310
Residual flaky	1.938	−0.0102^a^(H2)	0.708	0.174	0.417

*Notes*. TPA variables are abbreviated as: A, Adhesiveness; C1b, Chewiness‐1b; Co, Cohesiveness; H1, Hardness‐1; H1br, Residual Hardness‐1b; H1r, Residual Hardness‐1; H2, Hardness‐2; R, Resilience; Sr, Residual Springiness; T1, Thickness‐1; T2, Thickness‐2.

^a,b^Indicates that the coefficient have a probability of 0.01 and 0.05, respectively.

The IQF catfish data resulted in lower *R* ^2^s and slightly greater RMSEs than the fresh‐frozen catfish data although there were more IQF fish samples to create the models than there were fresh‐frozen catfish samples (Table 7). The *R* ^2^s ranged from 0.09 to 0.16. The RMSEs were less than 1.0, except residual firmness. The attribute, fibrous, had the greatest *R* ^2^ at 0.16, and its model included hardness‐1, and chewiness‐1. The cross‐products included (hardness‐1 × hardness‐1) and (chewiness‐1 × chewiness‐1). The moisture release and residual flaky predictor variables and coefficients had a slightly lower *R* ^2^ (0.14) and a slightly greater RMSE, with slightly less ability to predict them. Flaky had an *R* ^2^ less than the residual flaky at 0.12 and an RMSE of 0.86. The TPA parameters included were harness‐1b and chewiness‐1b. Firmness and residual firmness's stepwise regressions resulted in similar *R* ^2^s and RMSEs. The conventional firmness model included TPA hardness‐2 (from the second compression) and chewiness‐2, as well as the crosses (hardness‐2 × hardness‐2) and (hardness‐2 × chewiness‐2). The residual firmness included resilience and residual chewiness‐1 variables and the cross (resilience × residual chewiness‐1). Residual firmness did not include any of the TPA hardness parameters. The sensory attributes, cohesiveness of mass, and residual cohesiveness of mass, in the IQF catfish had *R* ^2^s less than 0.1, while moisture retention had no TPA parameters with correlations.

**Table 7:** Individually quick frozen processed fish equations that predict sensory texture from texture profile analysis (TPA) attributes

Sensory attribute	Intercept	TPA Predictor variable and coefficients	RMSE	*R* ^2^	*R*
Cohesiveness of mass	1.909	0.0043(H1)+6.512(Co) −0.00004(H1 × H1)+315.4(Co × Co)	0.698	0.094	0.307
Fibrous	4.188	0.0162^b^(H1) −0.0279(C1) −0.0003^a^(H1 × H1)+0.0015^b^(C1 × C1)	0.708	0.160	0.400
Firmness	2.566	0.0138(H2) −0.0128(C2) −0.0006(H2 × H2)+0.0013(H2 × C2)	0.866	0.102	0.319
Flaky	6.239	−0.0192(H1b)+0.0272(C1b)+0.00006(H1b × H1b)	0.862	0.124	0.352
Moisture release	5.577	23.32^a^(Cor)+274.3(Cor × Cor)	1.050	0.141	0.375
Moisture retention	—	No correlations	—	—	—
Springiness	1.284	0.0122^a^(H1br)	0.800	0.117	0.342
Residual cohesiveness of mass	0.0936	0.0160^b^(C1r)	0.704	0.069	0.263
Residual firmness	1.589	−0.0761(R)+0.0761^a^(C1r)+0.0047(R × C1r)	0.863	0.100	0.316
Residual flaky	−1.065	−0.0093(H1br)+0.1168(R) −0.0075(R × R)	0.842	0.141	0.375

*Notes*. TPA variables are abbreviated as: C1, Chewiness‐1; C1b, Chewiness‐1b; C1r, Residual Chewiness‐1; C2, Chewiness‐2; Co, Cohesiveness; Cor, Residual Cohesiveness; H1, Hardness‐1; H1b, Hardness‐1b; H1br, Residual Hardness‐1b; H2, Hardness‐2; R, Resilience.

^a,b^indicates that the coefficient have a probability of 0.01 and 0.05, respectively.

## 4 CONCLUSION

Catfish processed with the IQF processing method resulted in different intensities of sensory texture attributes to that found for fresh‐frozen catfish. The IQF fish had significantly more moisture release and moisture retention than fresh‐frozen catfish and were somewhat less firm than fresh‐frozen. The TPA evaluation of catfish was more successful than descriptive sensory texture analysis for identifying differences between IQF and fresh‐frozen catfish. Springiness, thickness‐1, and the residuals of springiness, chewiness‐1, and chewiness‐1b had *p *<* *0.001, and chewiness‐1, chewiness‐2, cohesiveness, hardness‐1, harndess‐2, resilience, residual cohesiveness, and residual harness‐1b all had *p *<* *0.05, while adhesiveness, chewiness‐1b, hardness‐1b, and thickness‐2 were not significantly different between IQF and fresh‐frozen catfish. All significant TPA parameters except cohesiveness, residual cohesiveness, and thickness‐1 were greater in the fresh‐frozen than IQF catfish.

Texture profile analysis was better able to predict sensory texture attributes in fresh‐frozen than in IQF catfish fillets with greater coefficients of determination (*R* ^2^) and slightly lower root mean square error (RMSE). In fresh‐frozen catfish, the sensory texture attributes, moisture retention, firmness, residual cohesiveness of mass, and flaky were predicted from TPA parameters with *R* ^2^s of ~0.25 or greater. In IQF catfish data, most sensory attributes were marginally predictable with TPA parameters.

## CONFLICT OF INTEREST

The authors declare that they do not have any conflict of interest. Mention of trade names or commercial products in this article is solely for the purpose of providing specific information and does not imply recommendation or endorsement by the U.S. Department of Agriculture.

## ETHICAL STATEMENTS

The United States Department of Agriculture, Research, Education and Economics established Policies and Procedures No. 605.1 on Protection of Human Subjects which complies with the Code of Federal Regulations (7 CFR 1c) governing protection of human subjects in research. Section 1c.101.b.6.i‐ii states the exemption from institutional review broads if food evaluation studies consist of wholesome foods without additives, or food that contains food ingredients at or below the level for use found to be safe, or agricultural chemical or environmental contaminant at or below the level found to be safe by the Food and Drug Administration or approved by the Environmental Protection Agency or the Food Safety and Inspection Service of the U.S. Department of Agriculture. The ARS‐SRRC sensory laboratory requires that each participating panelist sign an informed consent form before evaluating food samples. This form defines the food to be evaluated and the source.

No animal testing was performed in this study.

## References

[fsn3737-bib-0001] Ashton, T. J. , Michie, I. , & Johnston, I. A. (2010). A novel tensile test method to assess texture and gaping in salmon fillet. Journal of Food Science, 75, S182–S190. 10.1111/j.1750-3841.2010.01586.x 20546420

[fsn3737-bib-0002] Babyak, M. A. (2004). What you see may not be what you get: A brief, nontechnical introduction to overfitting in regression‐type models. Psychosomatic Medicine, 66, 411–421. 10.1097/01.psy.0000127692.23278.a9 15184705

[fsn3737-bib-0003] Barroso, M. , Careche, M. , & Borderias, A. J. (1998). Quality control of frozen fish using rheological techniques. Trends in Food Science and Technology, 9, 223–229. 10.1016/s0924-2244(98)00047-8

[fsn3737-bib-0004] Chambers IV, E. , & Robel, A. (1993). Sensory characteristics of selected species of freshwater fish in retail distribution. Journal of Food Science, 58, 508–512. 10.1111/j.1365-2621.1993.tb04312.x

[fsn3737-bib-0005] Cheng, L. , & Opara, U. L. (2013). Approaches to analysis and modeling texture in fresh and processed foods – A review. Journal Food Engineering, 119, 497–507. 10.1016/j.jfoodeng.2013.06.028.

[fsn3737-bib-0006] Cheng, J. , Sun, D. , Han, Z. , & Zeng, X. (2014). Texture and structure measurements and analyses for evaluation of fish and fillet freshness quality: A review. Comprehensive Reviews in Food Science and Food Safety, 13, 52–61. 10.1111/1541-4337.12043 33412693

[fsn3737-bib-0007] Cheret, R. , Delbarre‐Ladrat, C. , Lamballerie‐Anton, M. , & Verrez‐Bagnis, V. (2007). Calpain and cathepsin activities in post mortem fish and meat muscles. Food Chemistry, 101, 1474–1479. 10.1016/j.foodchem.2006.04.023

[fsn3737-bib-0008] Coppes, Z. , Pavlisko, A. , & De Vecchi, S. (2002). Texture measurements in fish and fish products. Journal Aquatic Food Product Technology, 11, 89–105. 10.1300/j030v11n01_08

[fsn3737-bib-0009] Crapo, C. , Himelbloom, B. , Pfutzenreuter, R. , & Lee, C. (1999). Texture modification processes for giant grenadier (*Albatrossia pectoralis*) fillets. Journal Aquatic Food Product Technology, 8, 27–40. 10.1300/j030v08n04

[fsn3737-bib-0010] DeMan, J. M. , & Kamel, B. S. (1981). Instrumental methods of measuring texture of poultry meat. Quality of poultry meat (pp. 157–164). Apeldoorn, The Netherlands: Spelderholt Jubilee Symposia.

[fsn3737-bib-0011] Drake, M. A. , & Gerard, P. D. (1999). Relationship between instrumental and sensory measurements of cheese texture. Journal of Texture Studies, 30, 451–476.

[fsn3737-bib-0012] Etemadian, Y. , Shabanpour, B. , Sadeghi Mahoonak, A. R. , Shabani, A. , & Alami, M. (2011). Cryoprotective effects of polyphosphates on *Rutilus frisii kutum* fillets during ice storage. Food Chemistry, 129, 1544–1551. 10.1016/j.foodchem.2011.06.005

[fsn3737-bib-0013] Griffiths, N. M. , & Wilkinson, C. C. L. (1978). The effects on broiler chicken of polyphosphate injection during commercial processing. II. Sensory assessment by consumers and an experienced panel. International Journal of Food Science & Technology, 13, 541–549. 10.1111/j.1365-2621.1978.tb00835.x

[fsn3737-bib-0014] Hale, M. B. , & Waters, M. E. (1981). Frozen storage stability of whole and headless freshwater prawns (*Macrobrachium rosenbergii*). Marine Fisheries Review, 42, 18–21.

[fsn3737-bib-0015] Hallier, A. , Chevallier, S. , Serot, T. , & Prost, C. (2007). Influence of farming conditions on colour and texture of European catfish (*Silurus glanis*) flesh. Journal of the Science of Food and Agriculture, 87, 814–823. 10.1002/jsfa.2779

[fsn3737-bib-0016] Hanson, T. , & Sites, D. (2015). 2014 U.S. Catfish Database. Available from http://www.agecon.msstate.edu/whatwedo/budgets/docs/catfish2014.pdf [last accessed July 2018].

[fsn3737-bib-0017] Hyldig, G. , & Nielsen, D. (2001). A review of sensory and instrumental methods used to evaluate the texture of fish muscle. Journal of Texture Studies, 32, 219–242. 10.1111/j.1745-4603.2001.tb01045.x

[fsn3737-bib-0018] Jiang, M. , Wang, Y. , van Santen, E. , & Chappel, J. A. (2008). Evaluation of textural properties of channel catfish (*Ictalurus punctatus* Rafinesque) fillet with the natural contour method. LWT – Food Science and Technology, 41, 1548–1554. 10.1016/j.lwt.2007.11.022

[fsn3737-bib-0019] Johnson, M. (2014). Overview of texture profile analysis. Available from http://www.texturetechnologies.com/resources/texture-profile-analysis [last accessed July 2018].

[fsn3737-bib-0020] Karales, S. (2001). Vacuum tumbling of meats and other foods. United States Patent US6040013A. 1‐5.

[fsn3737-bib-0021] Kin, S. , Schilling, M. W. , Smith, B. S. , Silva, J. L. , Jackson, V. , & Kim, T. J. (2010). Phosphate type affects the quality of injected catfish fillets. Journal of Food Science, 75, S74–S80. 10.1111/j.1750-3841.2009.01433.x 20492205

[fsn3737-bib-0022] Kin, S. , Schilling, M. W. , Smith, B. S. , Silva, J. L. , Kim, T. , Pham, A. J. , & Campano, S. G. (2011). Potassium acetate and potassium lactate enhance the microbiological and physical properties of marinated catfish fillets. Journal of Food Science, 76, S242–S250. 10.1111/j.1750-3841.2011.02122.x 22417369

[fsn3737-bib-0023] Klose, A. A. , Campbell, A. A. , & Hanson, H. L. (1963). Influence of polyphosphates in chilling water on quality of poultry meat. Poultry Science, 42, 743–749. 10.3382/ps.0420743

[fsn3737-bib-0024] Lampila, L. E. (1993). Functions and uses of phosphates in the seafood industry. Journal of Aquatic Food Product Technology, 1, 29–41. 10.1300/j030v01n03_04

[fsn3737-bib-0025] Li, C. H. , Bland, J. M. , & Bechtel, P. J. (2017). Effect of precooking and polyphosphate treatment on the quality of catfish fillets cooked in pouch in boiling water. International Journal of Food Science & Technology, 52, 1844–1851. 10.1111/ijfs.13459 PMC5448386 28572972

[fsn3737-bib-0026] Li, T. , Li, J. , Hu, W. , & Li, X. (2013). Quality enhancement in refrigerated red drum (*Sciaenops ocellatus*) fillets using chitosan coatings containing natural preservatives. Food Chemistry, 138, 821–826. 10.1016/j.foodchem.2012.11.092 23411183

[fsn3737-bib-0027] Li, T. , Li, J. , Hu, W. , Zhang, X. , Li, X. , & Zhao, J. (2012). Shelf‐life extension of crucian carp (*Carassius auratus*) using natural preservatives during chilled storage. Food Chemistry, 135, 140–145. 10.1016/j.foodchem.2012.04.115

[fsn3737-bib-0028] Lingqiao, M. A. , Chenglong, Q. I. , Jingjing, C. A. O. , & Dapeng, L. I. (2014). Comparative study on muscle texture profile and nutritional value of channel catfish (*Ictalurus punctatus*) reared in ponds and reservoir cages. Journal of Fishery Sciences of China, 38, 538–544.

[fsn3737-bib-0029] Lyon, B. G. , Champagne, E. T. , Vinyard, B. T. , & Windham, W. R. (2000). Sensory and instrumental relationships of texture of cooked rice from selected cultivars and postharvest handling practices. Cereal Chemistry, 77, 66–69. 10.1094/cchem.2000.77.1.64

[fsn3737-bib-0030] Lyon, B. G. , & Lyon, C. E. (1990). Texture profile of broiler pectoralis major as influenced by post‐mortem deboning time and heat method. Poultry Science, 69, 329–340. 10.3382/ps.0690329

[fsn3737-bib-0031] Lyon, B. G. , & Lyon, C. E. (1993). Effects of water cooking in heat‐sealed bags versus conveyor belt grilling on yield, moisture and texture of broiler breast meat. Poultry Science, 72, 2157–2165. 10.3382/ps.0722157

[fsn3737-bib-0032] Lyon, B. G. , & Lyon, C. E. (1997). Sensory descriptive profile relationships to shear values of deboned poultry. Journal of Food Science, 62, 885–897. 10.1111/j.1365-2621.1997.tb15479.x

[fsn3737-bib-0033] Meilgaard, M. , Civille, G. V. , & Carr, B. T. (2007). Sensory evaluation techniques. Chap. 9, p. 141‐172, Chap. 11, p. 189–253, 4th ed Boca Raton, FL: CRC Press.

[fsn3737-bib-0034] Meullenet, J. , Lyon, B. G. , Carpenter, J. A. , & Lyon, C. E. (1998). Relationship between sensory and instrumental texture profile attributes. Journal of Sensory Studies, 13, 77–93. 10.1111/j.1745-459x.1998.tb00076.x

[fsn3737-bib-0035] Montejano, J. G. , Hamann, D. D. , & Lanier, T. C. (1985). Comparison of two instrumental methods with sensory texture of protein gels. Journal of Texture Studies, 16, 403–424. 10.1111/j.1745-4603.1985.tb00705.x

[fsn3737-bib-0036] Moradi, Y. , Bakar, J. , Syed Muhamad, S. K. , & Che Man, Y. (2009). Effects of different final cooking methods on physico‐chemical properties of breaded fish fillets. American Journal of Food Technology, 4, 136–145. 10.3923/ajft.2009.136.145

[fsn3737-bib-0037] Morkore, T. , & Einen, O. (2003). Relating sensory and instrumental texture analyses of Atlantic salmon. Journal of Food Science, 68, 1492–1497. 10.1111/j.1365-2621.2003.tb09672.x

[fsn3737-bib-0038] Nakayama, T. , Hatae, K. , Kasai, M. , & Ooi, A. (2017). Seasonal changes in rigor development and flesh texture of wild Japanese Sea Bass (*Lateolabrax japonicas*). Journal of Aquatic Food Product Technology, 26, 578–592. 10.1080/10498850.2016.1240280

[fsn3737-bib-0039] Ojagh, S. M. , Shabanpour, B. , & Jamshidi, A. (2013). The effect of different pre‐fried temperatures on physical and chemical characteristics of silver carp fish (*Hypophthalmichthys molitrix*) nuggets. World Journal of Fish and Marine Sciences, 5, 414–420.

[fsn3737-bib-0040] Pascua, Y. , Koc, H. , & Foegeding, E. A. (2013). Food structure: Roles of mechanical properties and oral processing in determining sensory texture of soft materials. Current Opinion in Colloid and Interface Science, 18, 324–333. 10.1016/j.cocis.2013.03.009

[fsn3737-bib-0041] Sams, A. R. (1990). Electrical stimulation and high temperature conditioning of broiler carcasses. Poultry Science, 69, 1781–1786. 10.3382/ps.0691781

[fsn3737-bib-0042] Segars, R. A. , & Johnson, E. A. (1986). Instrumental measurement of the textural quality of fish flesh: Effect of pH and cooking temperature In Kramer D. E. & Liston J. (Eds), Seafood quality determination, Proceedings of an International Symposium Coordinated by the University of Alaska Sea Grant College Program, Anchorage, Alaska, USA, 10‐14 November. 49–61.

[fsn3737-bib-0043] Sheard, P. R. , Nute, G. R. , Richardson, R. I. , Perry, A. , & Taylor, A. A. (1999). Injection of water and polyphosphate into pork to improve juiciness and tenderness after cooking. Meat Science, 51, 371–376. 10.1016/s0309-1740(98)00136-3 22062033

[fsn3737-bib-0044] Sigurgisladottir, S. , Hafsteinsson, H. , Jonsson, A. , Lie, O. , Nortvedt, R. , Thomassen, M. , & Torrissen, O. (1999). Textural properties of raw salmon fillets as related to sampling method. Journal of Food Science, 64, 99–104. 10.1111/j.1365-2621.1999.tb09869.x

[fsn3737-bib-0045] Sigurgisladottir, S. , Tornissen, O. , Lie, O. , Thomassen, M. , & Hfsteinsson, H. (1997). Salmon quality: Methods to determine the quality parameters. Fisheries Science, 5, 223–252. 10.1080/10641269709388599

[fsn3737-bib-0046] Truong, V. D. , Daubert, C. R. , Drake, M. A. , & Baxter, S. R. (2002). Vane rheometry for textural characterization of cheddar cheeses: Correlation with other instrumental and sensory measurements. Lebensm‐Wiss U‐Technol, 35, 305–314. 10.1006/fstl.2001.0872

[fsn3737-bib-0047] Vacha, F. , Cepak, I. , Urbanek, M. , Vejsada, P. , Hartvich, P. , & Rost, M. (2013). Impact of long‐term storage on the instrumental textural properties of frozen common carp (*Cyprinus carpio*, L.) flesh. International Journal of Food Properties, 16, 241–250. 10.1080/10942912.2010.551309

[fsn3737-bib-0048] Veland, J. O. , & Torrissen, O. J. (1999). The texture of Atlantic salmon (*Salmo salar*) muscle as measured instrumentally using TPA and Warner‐Brazler shear test. Journal of the Science of Food and Agriculture, 79, 1737–1746. 10.1002/(sici)1097-0010(199909)79:12<1737:aid-jsfa432>3.0.co;2-y

[fsn3737-bib-0400] Woyewoda, A. D. , & Bligh, E. G. (1986). Effect of phosphate blends on stability of cod fillets in frozen storage. Journal of Food Science, 51, 932–935. 10.1111/j.1365-2621.1986.tb11202.x

